# Absolute Negative Mobility of Inertial Active Ornstein–Uhlenbeck Particles in Media with Power-Law Correlated Noise

**DOI:** 10.3390/e28101047

**Published:** 2026-09-24

**Authors:** Shengna Liu, Kheder Suleiman

**Affiliations:** 1 School of Mathematics and Statistics, Henan University of Technology, Zhengzhou 450001, China; t2024011@haut.edu.cn; 2 School of Mathematics and Statistics, Northwestern Polytechnical University, Xi’an 710072, China

**Keywords:** anomalous transport, negative mobility, active particles, fractional brownian motion, viscoelastic media

## Abstract

This study investigates the transport properties of inertial Active Ornstein–Uhlenbeck particles (AOUPs) in a one-dimensional rocking washboard potential externally imposed fractional Gaussian noise (fGn). Through numerical simulations, we explore how fGn in the potential influences particle mobility. Our findings reveal anomalous transport, including absolute negative mobility (ANM) at specific active intensity levels. The Hurst exponent significantly modulates ANM, with higher values of the Hurst exponent widening the ANM window by reducing the noise’s anti-persistence (all Hurst exponents studied here satisfy H≤1/2). These results provide new insights into the mechanisms governing anomalous transport in media with power-law correlated noise. Our findings have potential applications in microfluidics and active matter systems, enabling precise manipulation and separation of active particles.

## 1. Introduction

Self-propelled particles are entities capable of converting stored or ambient energy into directed motion, distinguishing them from passive particles driven by external forces like fluid flow or thermal noise from collisions with surrounding molecules. These particles exhibit a unique blend of random and persistent motion, transitioning from short-time ballistic motion to long-time enhanced diffusion in homogeneous environments at low Reynolds numbers as their activity decreases [1]. They are broadly categorized into two types: wet self-propelled particles, such as swimming bacteria and motile cells, which rely on metabolic processes for propulsion [2,3,4,5], and dry self-propelled particles, like microrobots and active Janus particles, which move through mechanisms such as shape deformation or phoresis in response to electric fields, chemotactic cues, or temperature gradients [6,7,8]. Over the past two decades, self-propelled particles have garnered significant interest in physics and biology due to their emergent collective behaviors—such as flocking, clustering, phase separation, and spontaneous flows—that are absent in passive particle systems [9,10,11,12,13,14,15]. Moreover, their potential applications extend to fields such as gene therapy and targeted drug delivery in medicine [16,17,18].

Active Ornstein–Uhlenbeck particles (AOUPs) [19,20,21] and Run-and-Tumble particles (RTPs) [22,23,24] are two of the most widely used minimal stochastic models for self-propelled active particles. While RTPs describe motion through alternating straight runs and random tumbling events, AOUPs represent self-propulsion by an Ornstein–Uhlenbeck process with finite temporal correlations. Both models successfully capture the persistent nature of active motion and have been extensively employed to investigate nonequilibrium transport, diffusion, and collective phenomena in active matter [19,20,25,26,27,28,29,30,31,32,33,34]. In the present work, we focus exclusively on the AOUP model. Here, the active force evolves according to an Ornstein–Uhlenbeck process characterized by a finite correlation time τ (the persistence time) and an activity strength $D_{ac}$ [19,20,21]. The finite temporal correlation of the active force captures the persistence of self-propelled motion while retaining a mathematically tractable description. Owing to its simplicity and analytical accessibility, the AOUP model has become a standard framework for studying transport processes in active matter, particularly for synthetic microswimmers and active colloidal systems [35,36,37,38].

The response of particles to external forces is a key area of study in statistical physics, particularly when systems are driven far from equilibrium. According to the Le Chatelier-Braun principle, a system in thermal equilibrium adjusts to an external force $f_{0}$ by aligning with it, typically increasing velocity *v* proportionally for small forces [39,40]. However, this relationship between the applied force and the corresponding velocity can exhibit remarkable anomalies, particularly as the system moves far from equilibrium. These anomalies include negative differential mobility [41,42,43,44], in which the velocity decreases with increasing force, and absolute negative mobility (ANM), where the velocity not only decreases with an increase in applied force but exhibits an opposite sign [45]. ANM has been observed in diverse systems, including semiconductor heterostructures, colloidal suspensions, and ultracold atoms in optical lattices [46,47,48,49,50,51,52,53,54,55,56,57]. This phenomenon often arises from complex interactions between external forces, internal dissipation, and system dynamics, offering potential for precise control in applications like microfluidic particle sorting [53,58].

These realizations have spurred extensive research in this field, with various approaches explored to model such an environment [39,40,52,59,60,61,62,63]. In this context, for a passive tracer particle, two essential criteria must be met for the emergence of ANM. First, the system must be driven significantly away from its thermal equilibrium, resulting in a dynamic, time-dependent non-equilibrium condition. Second, the ensuing dynamics should display an unbiased average response that tends toward zero under non-equilibrium conditions. In their study on Hamiltonian coupled transport, Wang et al. [64] demonstrate the emergence of inverse currents when coupled thermodynamic forces perturb the equilibrium state. Understanding and exploitation of this intriguing effect have led to the development of specialized microfluidic devices capable of efficiently sorting micrometric particles [52,53,58], with further advancements promising even more precise control over the transport of small-scale objects.

While ANM is well-documented in passive systems, its exploration in active particle systems remains limited [65,66,67,68,69,70]. This gap is particularly pronounced in complex environments like cellular cytoplasm, where periodic potentials with fractional Gaussian noise (noise with power-law temporal correlations) are prevalent [71,72,73,74]. This work numerically investigates ANM in AOUPs within a rocked tilted potential, examining the role of fGn. The oscillating external force provides periodic energy input, destabilizing locked states, while self-propulsion introduces persistent motion that can reverse directionality. Power-law correlated (nonequilibrium) noise with 0<α≤1 reflects anomalous diffusion in crowded media [72,73], introducing memory effects that modulate ANM. These components are complementary, tuning the system for robust anomalous transport.

Although overdamped dynamics are standard for many wet active systems at low Reynolds numbers [2,3,4,5], recent studies highlight the importance of inertial (underdamped) effects in active matter, particularly for dry particles where momentum carry-over influences persistence and transport [75,76,77]. For instance, inertial extensions of the active Ornstein–Uhlenbeck model reveal modified diffusion and clustering behaviors due to finite mass [75,77], while experimental realizations in nanoscale systems demonstrate inertia’s role in propulsion dynamics [76]. In collective settings, inertia can drive emergent phenomena, such as phase separations and differential negative mobility, as shown in simulations of inertial active particles [78,79]. These findings motivate our inclusion of inertia to capture realistic transport anomalies in underdamped active regimes, relevant to applications like microfluidics and microrobotics [6,7,8,56].

The paper is structured as follows: Section 2 describes the stochastic dynamical system and relevant equations. The results are presented, analyzed, and discussed in Section 3. Specifically, the numerical results are analyzed based on the averaged velocity 〈v〉 and mobility $\langle v\rangle /f_{0}$ in Section 3.1 and Section 3.2, addressing the interplay of inertia, self-propulsion, and noise correlations. In Section 4, we discuss our results and conclude the study in Section 5.

## 2. The Model

We consider a one-dimensional underdamped self-propelled particle of mass *m* moving in a fluid with friction coefficient Γ, and subjected to the rocked tilted potential. The dynamics are governed by the stochastic differential equation:

(1) $$m\ddot{x}\left(t\right)+\Gamma \dot{x}\left(t\right)=-V^{\prime }\left(x\right)+A\cos\left(\Omega t\right)+F_{0}+\xi_{ac}\left(t\right)+\sqrt{2\Gamma k_{\alpha }T}\xi_{\alpha }\left(t\right),$$

where $\ddot{x}\left(t\right)$ and $\dot{x}\left(t\right)$ are the second and first time derivatives of position *x*, $V^{\prime }\left(x\right)=\frac{\partial V(x)}{\partial x}$ is the spatial derivative of the symmetric periodic potential $V\left(x\right)=V_{0}\sin\left(\frac{2\pi x}{L}\right)$ with $V_{0}$ the amplitude of the potential (the peak-to-trough barrier is therefore $2V_{0}$) and *L* is the period, Acos(Ωt) is the rocking force with amplitude *A* and frequency Ω, and $F_{0}$ is a constant bias force. The noise term $\xi_{ac}\left(t\right)$ and $\xi_{\alpha }\left(t\right);\;0<\alpha <1$ represent active and anti-persistence colored noise, respectively. $\xi_{\alpha }\left(t\right)$ has zero mean and autocorrelation $\langle \xi_{\alpha }\left(t_{1}\right)\xi_{\alpha }\left(t_{2}\right)\rangle =\alpha \left(\alpha -1\right)|t_{1}-t_{2}{|}^{\alpha -2}+2\alpha {\left|t_{1}-t_{2}\right|}^{\alpha -1}\delta \left(t_{1}-t_{2}\right)$, where 0<α≤1. α=1 corresponds to white Gaussian noise (wGn) in which $k_{1}=k_{B}$ is the Boltzmann constant, while 0<α<1 corresponds to fGn where $k_{\alpha }$ is the generalized diffusion constant [73].

We emphasize that this correlation function is a formal, distributional definition, not a pointwise one, and we fix its interpretation here so that it is used consistently throughout the paper. Operationally, $\xi_{\alpha }\left(t\right)\equiv {\dot{B}}_{\alpha }\left(t\right)$ is defined as the derivative, in the sense of generalized functions, of a fractional Brownian motion $B_{\alpha }\left(t\right)$ with Hurst exponent H=α/2∈(0,1/2] (Mandelbrot–Van Ness); this fBm-based definition, not the correlation function above, is what is actually simulated. The coincident-time term $|t_{1}-t_{2}{|}^{\alpha -1}\delta \left(t_{1}-t_{2}\right)$ is the formal completion of the correlation that arises from differentiating the fBm covariance twice in the sense of distributions; it is a statement about $t_{1}=t_{2}$ only and plays no role for $t_{1}\neq t_{2}$. The pairwise correlation actually used in the analysis, quoted below Equation (3), is simply the expression above restricted to $t_{1}\neq t_{2}$, where it reduces to $\alpha \left(\alpha -1\right)|t_{1}-t_{2}{|}^{\alpha -2}$; the two expressions are therefore consistent, and the delta term does not need to be resolved for anything computed in this paper. Likewise, the numerical implementation (Appendix A) is built directly from $\xi_{\alpha }={\dot{B}}_{\alpha }$ as increments of a simulated fractional Brownian motion, and never evaluates the coincident-time term.

We stress that both $\xi_{\alpha }\left(t\right)$ and $\xi_{ac}\left(t\right)$ are *external* noise sources: the friction coefficient Γ in Equation (1) is a fixed, memoryless (instantaneous) drag that is not tied to the autocorrelation of either noise. Consequently the second fluctuation–dissipation theorem is not imposed and is, in general, not satisfied: this is manifestly true for the active noise $\xi_{ac}\left(t\right)$ at all τ, and for $\xi_{\alpha }\left(t\right)$ whenever α<1. Only in the limit α=1, $D_{ac}=0$ does Equation (1) reduce to a particle in genuine equilibrium with a thermal bath at temperature *T*; for α<1 we accordingly refer to $\xi_{\alpha }\left(t\right)$ as an externally imposed non-equilibrium colored noise representing, e.g., the effect of a crowded viscoelastic medium, rather than as an equilibrium “thermal” fluctuation. A genuine equilibrium generalized Langevin description of such a medium would instead require a memory-friction kernel related to $\langle \xi_{\alpha }\left(t_{1}\right)\xi_{\alpha }\left(t_{2}\right)\rangle$ through the second fluctuation–dissipation theorem, which is not what is implemented here.

For AOUPs, $\xi_{ac}\left(t\right)$ follows the Ornstein–Uhlenbeck process:

(2) $${\dot{\xi }}_{ac}\left(t\right)=-\tau^{-1}\xi_{ac}\left(t\right)+\sqrt{2D_{ac}\tau^{-1}}\zeta \left(t\right),$$

with ζ(t) as white Gaussian noise (wGn). In this context, the autocorrelation function $\langle \xi_{ac}\left(t_{1}\right)\xi_{ac}\left(t_{2}\right)\rangle =D_{ac}\exp(-|t_{1}-t_{2}\left|/\tau \right)$ where τ is the persistence time and $D_{ac}$ activity intensity.

It is instructive to relate Equation (1) to several well-established models that are recovered in appropriate limiting cases. When the rocking force, constant bias, and active noise are absent ($a=f_{0}=0$, $\xi_{\mathrm{ac}}=0$), and α=1, Equation (1) reduces to the classical underdamped Langevin equation describing a Brownian particle moving in a periodic potential. When the active noise is switched off ($D_{\mathrm{ac}}=v_{0}=0$) while retaining the rocking force and Gaussian white noise (α=1), the model reduces to the inertial rocked washboard system in which absolute negative mobility (ANM) induced by thermal equilibrium fluctuations was first demonstrated [39,61]. This limiting case constitutes the direct parent model of the present work, and the deterministic and thermal-only results shown in Figure 1 reproduce the established behavior, thereby providing a benchmark for our numerical implementation. In the absence of the rocking force (a=0), with a constant bias ($F_{0}\neq 0$) and active noise, Equation (1) reduces to the standard AOUPs transport model in tilted periodic potentials [19,23]. The present model therefore provides a unified framework that extends these previously studied limits by simultaneously incorporating active self-propulsion and externally imposed fractional Gaussian noise into the classical rocked washboard geometry.

We now rewrite the underdamped Equation (1) in dimensionless form using the characteristic scales: $\tilde{x}=x/L$, $\tau_{0}=\sqrt{mL^{2}/V_{0}}$, $\tilde{t}=t/\tau_{0}$, $\gamma =\Gamma \tau_{0}/m$, $a=LA/V_{0}$, $\omega =\Omega \tau_{0}$, $f_{0}=LF_{0}/V_{0}$, $D_{\alpha }=k_{\alpha }T/V_{0}$, and ${\tilde{D}}_{ac}=L^{2}D_{ac}/V_{0}^{2}$, where the time scale $\tau_{0}$ can be viewed as the characteristic period of oscillations inside the potential wells. Thus, the Equation (1) has the form:

(3) $$\ddot{\tilde{x}}+\gamma \dot{\tilde{x}}=-{\tilde{V}}^{\prime }\left(\tilde{x}\right)+a\cos\left(\omega t\right)+f_{0}+\xi_{ac}\left(t\right)+\sqrt{2\gamma D_{\alpha }}\xi_{\alpha }\left(t\right).$$

The dimensionless potential, defined as ${\tilde{V}}^{\prime }\left(\tilde{x}\right)=V\left(L\tilde{x}\right)/V_{0}$, exhibits a periodicity of L=1. The rescaled non-equilibrium colored noise is expressed as ${\tilde{\xi }}_{\alpha }\left(\tilde{t}\right)=L\xi_{\alpha }\left(t\right)/V_{0}$ and retains the same statistical characteristics as $\xi_{\alpha }$, namely $\langle {\tilde{\xi }}_{\alpha }\left(\tilde{t}\right)\rangle =0$ and, for ${\tilde{t}}_{1}\neq {\tilde{t}}_{2}$, $\langle {\tilde{\xi }}_{\alpha }\left({\tilde{t}}_{1}\right){\tilde{\xi }}_{\alpha }\left({\tilde{t}}_{2}\right)\rangle =\alpha \left(\alpha -1\right){\left|{\tilde{t}}_{1}-{\tilde{t}}_{2}\right|}^{\alpha -2}$ (the coincident-time term ${\tilde{t}}_{1}={\tilde{t}}_{2}$ is discussed after Equation (1): it is a distributional statement about the diagonal only, so it is correctly absent here rather than inconsistently dropped—this expression is Equation (1) restricted to ${\tilde{t}}_{1}\neq {\tilde{t}}_{2}$, not a separate or conflicting definition). Likewise, the rescaled active process is given by ${\tilde{\xi }}_{ac}\left(\tilde{t}\right)=L\xi_{ac}\left(t\right)/V_{0}$ and shares the statistical properties of $\xi_{ac}$, such that $\langle {\tilde{\xi }}_{ac}\left({\tilde{t}}_{1}\right){\tilde{\xi }}_{ac}\left({\tilde{t}}_{2}\right)\rangle ={\tilde{D}}_{ac}\exp(-|{\tilde{t}}_{1}-{\tilde{t}}_{2}\left|/\tau \right)$. Moving forward, we will consistently use these dimensionless variables. To streamline the notation, the ˜ symbol will be omitted in the referenced equation, Equation (3). The system is solved numerically using an explicit Euler–Maruyama scheme for the velocity and active-noise variables, combined with a trapezoidal update for position, with Δt=0.01. For more details, see Appendix A.

The directed transport of the particle is quantified by the asymptotic averaged velocity

(4) $$\langle v\rangle \;=\;\lim_{M\to \infty }\frac{1}{MT}\;E\;\left[x\left(t_{tr}+MT\right)-x\left(t_{tr}\right)\right]\;=\;\lim_{M\to \infty }\frac{1}{MT}\int_{t_{tr}}^{t_{tr}+MT}E\left[\dot{x}\left(s\right)\right]\;ds,$$

where T=2π/ω is the period of the external drive, $t_{tr}$ is a transient time discarded to remove initial-condition dependence, M∈N is the number of driving periods over which the average is taken, and E[·] denotes an average over *N* independent realizations of the stochastic forcing (fGn/wGn and/or active noise). Equation (4) is thus simultaneously an ensemble average (over trajectories) and a time average (over *M* driving periods); in the asymptotic regime the two coincide by ergodicity of the periodically-driven process. In practice 〈v〉 is evaluated from the discretized trajectories as

(5) $$\langle v\rangle \;\approx \;\frac{1}{N}\sum_{i=1}^{N}\frac{1}{MT}\left[x_{i}\left(t_{tr}+MT\right)-x_{i}\left(t_{tr}\right)\right],$$

and we have verified (Appendix A) that this displacement-based estimator agrees with the equivalent estimator built from the time-averaged instantaneous velocity $\dot{x}\left(t\right)$, as required by Equation (4). All trajectories are initialized at x(0)=0, $\dot{x}\left(0\right)=0$, with the drive phase fixed at ωt=0; $t_{tr}$ is chosen, separately for each noise model, from the convergence analysis described in Appendix A.

We define absolute negative mobility (ANM) as the regime in which the averaged velocity is directed opposite to the constant symmetry-breaking bias:

(6) $$\mathrm{ANM}\;⟺\;\mathrm{sgn}\left(\langle v\rangle \right)=-\;\mathrm{sgn}\left(f_{0}\right),\;f_{0}\neq 0.$$

This is distinct from, and should not be confused with, motion relative to the zero-mean rocking force acos(ωt): since this term averages to zero over one period, it cannot by itself define a preferred direction, and does not constitute the symmetry-breaking field against which ANM is defined. The rocking force instead plays the role of the non-equilibrium drive that, together with the noise sources considered here, enables the symmetry breaking responsible for ANM at $f_{0}\neq 0$.

For the spatially symmetric system (3), Equation (6) implies the antisymmetry

(7) $$\langle v\rangle \left(-f_{0}\right)=-\;\langle v\rangle \left(f_{0}\right),$$

which follows directly from the odd symmetry of the periodic potential, U(−x)=−U(x), under the combined transformation x→−x, $f_{0}\to -f_{0}$, together with the symmetric distribution of the noise sources used here. We verify Equation (7) numerically in Appendix A (Figure A3).

## 3. Results

In this work, the term *anomalous transport* refers specifically to the phenomenon of absolute negative mobility (ANM), whereby the stationary average velocity is opposite in sign to the applied constant bias despite the external driving. Although the term *anomalous diffusion* is commonly used to describe deviations from normal Brownian diffusion, characterized by a non-linear time dependence of the mean-squared displacement, it is not the focus of the present study. To avoid ambiguity, the term “anomalous” is used throughout this paper exclusively in the context of anomalous transport.

### 3.1. Absolute Negative Mobility for Active Particles

In the context of the rocked tilted washboard periodic potential described in Equation (1), motion can be confined to a finite number of spatial periods or can extend infinitely, as shown in Figure 1a. This unbounded motion is particularly important for deterministic behavior. The velocity of unbounded motion indicates the direction of the bias force, $f_{0}$. However, as illustrated in the same panel, there are certain states where a≫3.9 that exhibit unbounded motion averaging in the opposite direction of the constant driving force $f_{0}$. This phenomenon demonstrates deterministic absolute negative mobility (ANM). In these deterministic cases, the energy needed to move upward is supplied by the oscillating driving force.

The existence of thermal fluctuations, represented as $\xi_{\alpha }$ where α=1, results in stochastic transitions between coexisting potential basins, as depicted in Figure 1b. This process serves a role analogous to that of potential wells in systems at equilibrium. Additionally, these fluctuations can destabilize confined states, facilitating transitions between adjacent states that lead to either diffusive motion or direct transport. Such behavior can induce the ANM effect under certain parameter conditions, as illustrated in Figure 2a when $D_{ac}=0$. Here, it is observed that the average velocity diminishes across a range of forces $f_{0}$ near zero [39]. Interestingly, even a slight introduction of thermal noise can result in negative average velocity at low applied forces. The boundaries for ANM are marked by stall forces $\pm f_{\mathrm{stall}}$, where the average velocities drop to zero, as shown in Figure 2a. As the temperature rises, the stall force decreases, ultimately vanishing at a certain finite temperature [39].

The ANM discussed earlier arises solely in the context of thermal fluctuations; however, the deterministic dynamics of the system play a vital role in shaping the mechanisms behind it. When the driving strength is set to a=4.2, the deterministic system features a single stable period-1 orbit, a locked state where the particle oscillates within one potential well, resulting in an average velocity of zero (〈v〉=0). This is the baseline state without noise, as confirmed by numerical integration of Equation (3) with $\xi_{\alpha }=\xi_{ac}=0$. Figure 2a shows how 〈v〉 remains zero at $f_{0}=0$ in the absence of noise, transitioning to negative values with active noise (e.g., $D_{ac}=0.001$), illustrating the destabilization of this locked state. Nevertheless, numerous unstable periodic orbits exist that can propel the particle in both positive and negative directions, influencing the relaxation dynamics when the system is initialized far from the stable locked orbit. When noise is introduced, the particle is frequently disturbed away from the stable periodic orbit. For a=4.2, there is an optimal temperature at which ANM becomes most prominent, as indicated in Figure 2a. In contrast, for a=5.1, increasing the temperature eventually suppresses ANM entirely. For a=4.4, an optimal temperature arises where 〈v〉 reaches its maximum value.

At $D_{ac}=0$, Figure 2a already shows a clear thermal ANM window, as established in the preceding paragraphs. Switching on the Ornstein–Uhlenbeck active noise $\xi_{ac}$ does not create this window; rather, it modulates the pre-existing passive (thermal-noise-only) ANM regime, as illustrated in Figure 2. In this context, the average velocity exhibits negative values in the region $0<f_{0}<0.3$ for $0<D_{ac}<0.01$, as seen in Figure 2a, i.e., activity extends the same sign-reversed-transport regime that is already present at $D_{ac}=0$. In contrast, for larger values of $f_{0}$ and noise intensity $D_{ac}$, the transport consistently aligns with the direction of the bias, so that sufficiently strong activity eventually destroys rather than induces ANM. We have specifically searched the $\left(f_{0},D_{ac}\right)$ plane for a parameter range in which the passive ($D_{ac}=0$) current is nonnegative within error bars and becomes statistically significantly negative upon increasing $D_{ac}$ from zero; we did not find such a region with the present data, and we do not claim activity-induced sign reversal.

To better understand this behavior, we present the time evolution of the displacement *x* and the velocities as a function of the amplitude *a* for AOUPs at varying intensities $D_{ac}$, as illustrated in Figure 3. Similarly to equilibrium thermal noise, active noise contributes to destabilizing the stable locked state; however, the extent and direction of this destabilization are influenced by the intensity $D_{ac}$. At low intensities, as depicted in Figure 3a with $D_{ac}=0.001$, most velocities exhibit negative values. In addition, Figure 3d,e reveal a consistent trend where the particle position shifts in the negative direction. During intervals between bursts of noise, periods of nearly regular motion occur, during which a negative average velocity is consistently observed, significantly influencing the occurrence of negative mobility. In contrast, at higher intensity levels, the locked state becomes destabilized, resulting in the majority of velocities becoming positive, as shown in Figure 3c for $D_{ac}=0.1$. This can be interpreted as transport that occurs in the direction of the bias $f_{0}$, as indicated in Figure 2, rather than against it.

The maximum values of $f_{0}$ and $D_{ac}$ at which the particles continue to move against the bias are influenced by $D_{ac}$ and $f_{0}$, respectively, with only a minor dependence on the propulsion memory time τ. Specifically, as the intensity $D_{ac}$ increases, the maximum allowable value of $f_{0}$ decreases, and vice versa. This relationship is illustrated in Figure 2b for τ=1. Furthermore, for smaller values of τ, both parameters tend to decrease, while the system exhibits more pronounced negative mobility for larger values of τ, as depicted in Figure 2b,c.

### 3.2. Effects of Fractional Gaussian Noise on ANM

Fractional Gaussian noise with Hurst exponent H=α/2 introduces memory effects, reflecting anomalous diffusion in crowded media [71,72,73]. Its role is illustrated in Figure 4, which displays the average velocity of AOUPs governed by the stochastic dynamical system (3) with varying values of *H*. The net motion against $f_{0}$ can persist in the presence of active noise and fGn. However, as α=2H decreases, the region $\left(f_{1},f_{2}\right)$ where active noise manifests ANM shrinks, and the maximum absolute value of the average velocity for AOUPs decreases, as demonstrated in Figure 4a. In contrast, the maximum intensity $D_{ac}$ at which ANM continues to emerge increases with the exponent α for AOUPs, as shown in Figure 4b. Thus, we conclude that the α exponent of fGn and the intensity $D_{ac}$ of active noise, regardless of its type, can serve as controlling parameters for ANM. These parameters can be adjusted to fine-tune the ANM regime for this active particle type and influence their directional movement. Within the range H∈(0,1/2] studied here, higher α (i.e., *H* closer to 1/2) corresponds to a *reduction* of anti-persistence—not an increase in persistence in the H>1/2 sense—and this reduced anti-persistence is associated with a wider ANM window. This memory-driven effect, absent in white noise, is crucial for stabilizing negative transport, with experimental relevance in biological systems [72].

## 4. Discussion

In this study, we investigated the transport properties of inertial active particles in a one-dimensional dynamic periodic potential under both thermal and non-equilibrium fluctuations, focusing on Active Ornstein–Uhlenbeck Particles (AOUPs). Our numerical analysis revealed a rich phenomenology of anomalous transport, with a key finding being the emergence of absolute negative mobility (ANM), where the long-time averaged velocity is opposite in sign to the applied constant bias $f_{0}$.

We provide below a focused discussion on the two driving mechanisms (oscillating external force and self-propulsion), the inclusion of inertia (underdamped dynamics), and the nonstandard noise correlations. We clarify their interplay, justifying their inclusion based on physical contexts and numerical findings. These elements are complementary: the oscillating force provides periodic energy input to destabilize locked states, while self-propulsion introduces persistent motion that modulates transport directionality. Inertia and correlated noise further tune the dynamics in regimes relevant to certain active systems (e.g., microrobots or cellular environments).

Inertia (modeled via the underdamped Langevin equation with mass m=1) is not a minimal condition for absolute negative mobility (ANM): the phenomenon has been observed in overdamped active systems, including Janus particles in corrugated channels [68] and, more directly relevant to the active-noise setting considered here, a one-dimensional overdamped system driven by active fluctuations [80]. Inertia does, however, qualitatively alter ANM in our setup by introducing oscillatory relaxation dynamics around potential minima, which we interpret as amplifying the destabilization of locked states under rocking forces and active noise, though we have not established this picture through an explicit dynamical-systems analysis (see below), nor have we performed the systematic mass/friction (m→0) sweep that a direct underdamped-versus-overdamped comparison within the present model would require. In the overdamped limit (m→0 or high friction γ), velocity adjusts instantaneously to forces, reducing the window for negative response; our underdamped regime allows inertia to “carry” particles against the bias during transient phases, leading to stronger ANM at low active intensities (e.g., $D_{ac}<0.01$, as in Figure 2a). We stress that this contrast is drawn qualitatively against the overdamped ANM literature rather than from a controlled overdamped limit of the present model; a systematic scan over mass and friction connecting the underdamped and overdamped regimes of Equation (3) is left for future work. We include inertia to model “dry” active particles like microrobots [6,7,8], where low-Reynolds assumptions do not always hold, unlike purely overdamped wet systems [2,3,4,5].

Self-propulsion, via active noise $\xi_{ac}\left(t\right)$, is plausibly consistent with destabilizing stable locked orbits in the rocked potential, offering one physical interpretation of how transport is promoted against the bias $f_{0}$ at low intensities (e.g., $0<D_{ac}<0.01$; Figure 2). It acts as an internal energy source complementary to the external oscillating force, and this same destabilization picture would enable reversal by introducing persistent motion that competes with the bias during low-activity “quiet” periods (Figure 3d,e). At higher intensities, it suppresses ANM by overly randomizing trajectories, aligning motion with $f_{0}$. As noted in Section 3.1, however, this modulation acts on a passive ANM window already present at $D_{ac}=0$; we do not find evidence, within the present data, that activity by itself reverses an otherwise nonnegative passive current. AOUP noise (smooth Ornstein–Uhlenbeck noise, modeling dry colloids [19,20,21]) thus offers a controlled, analytically tractable route to activity-*modulated* ANM; extending this analysis to other active-noise statistics, such as run-and-tumble dynamics with discrete directional switching, and performing a systematic comparison based on matched persistence and active diffusivity, is an interesting direction for future work. We emphasize that the locked-orbit and destabilization language used throughout this section describes a plausible physical interpretation of the observed transport reversal, rather than an established dynamical mechanism: a rigorous dynamical-systems analysis, including the computation of the underlying periodic orbits, their stability, and the associated basins of attraction, lies beyond the scope of the present work and is left for future study.

The externally imposed, power-law correlated noise ($\langle \xi_{\alpha }\left(t_{1}\right)\xi_{\alpha }\left(t_{2}\right)\rangle =\alpha D_{\alpha }\left(\alpha -1\right)|t_{1}-t_{2}{|}^{\alpha -2}+2\alpha D_{\alpha }{\left|t_{1}-t_{2}\right|}^{\alpha -1}\delta \left(t_{1}-t_{2}\right)$, with 0<α≤1, and interpreted as in Section 2) physically represents anomalous diffusion in crowded, viscoelastic media like cellular cytoplasm [72,73], where molecular collisions create power-law temporal correlations (anti-persistent for Hurst exponent H=α/2<1/2). Unlike delta-correlated white noise (α=1), which lacks memory and models equilibrium Brownian motion, this fGn introduces non-Markovian effects: power-law decay promotes subdiffusive trapping and anti-persistence over the range studied here. This is crucial for ANM, as higher α—corresponding to reduced, not increased, anti-persistence within the range H≤1/2 studied here—widens the ANM regime by reducing erratic reversals (Figure 4; the maximum $D_{ac}$ for which ANM is observed increases with α). Experimentally, such noise arises in biological [72] or disordered systems [71,73], differing from white noise by fostering memory-driven anomalies that stabilize negative transport. We reiterate (Section 2) that, being external, this noise does not satisfy the second fluctuation–dissipation theorem for α<1; a genuine equilibrium viscoelastic bath would instead require a generalized Langevin equation with a matching memory-friction kernel.

In summary, the oscillating force drives baseline rocking, self-propulsion is associated with directional reversal via persistence, inertia is associated with oscillatory escapes, and correlated noise introduces memory to tune the ANM regime—all interplaying to produce robust anomalous transport in complex environments. These components are justified for applications in microfluidics [53,58], where parameter tuning enables particle sorting.

The minimal conditions for ANM in the present model include: (1) a non-equilibrium drive (ac rocking) to create time-dependent attractors, (2) an unbiased average response (〈v〉=0 at $f_{0}=0$) enabling symmetry breaking by noise, and (3) stochastic perturbations (thermal or non-equilibrium) to destabilize locked states. Inertia is not included in this minimal list: as noted above, ANM is known to occur in overdamped active systems as well [68,80], so underdamped dynamics should be regarded as a feature of the present setup that modulates the strength and location of the ANM window, not as a requirement for ANM itself. Threshold behavior shows an abrupt onset below critical noise intensities: ANM requires $0<D_{ac}<0.01$ and $0<f_{0}<0.3$ (Table A1 and Figure A1 and Figure A2 give the corresponding converged, error-barred estimates of $\bar{v}$ at representative points). Lower α shifts the onset to smaller intensities.

Key parameters controlling onset and magnitude include: active intensity ($D_{ac}$), with optimal low values; persistence time (τ), enhancing coherence; the Hurst exponent (H=α/2), widening the ANM regime as it approaches 1/2 from below within the range studied; rocking parameters (*a*, ω), tuning the attractor landscape; and friction (γ) and bias ($f_{0}$), favoring ANM at low values.

Finally, we comment on the possibility of stochastic resonance (SR) in this model, as the rocked washboard potential combined with the periodic drive acos(ωt) is a canonical setting for this phenomenon [81]. A rigorous linear-response/susceptibility analysis for the present underdamped, multiply-driven system is complicated by the fact that, as discussed in Section 2, our external fGn and active noises do not enter a fluctuation–dissipation relation with the friction. The optimal-intensity maxima already visible in our 〈v〉 vs. $D_{ac}$ and 〈v〉 vs. α scans (Section 3.1 and Section 3.2) are consistent with, though not proof of, an SR-like mechanism; a dedicated susceptibility/SR analysis, scanning ω at fixed noise intensity and computing the power spectrum of x(t), is left for future work.

## 5. Conclusions

In this study, we investigated the transport properties of inertial Active Ornstein–Uhlenbeck particles (AOUPs) moving in a one-dimensional rocked washboard potential subject to active fluctuations and externally imposed fractional Gaussian noise. We examined how the statistical properties of this active fluctuation, together with the correlated external noise, influence the emergence of absolute negative mobility (ANM).

Our numerical results demonstrate that ANM can arise over a broad range of parameters, where the long-time averaged velocity is opposite in sign to the applied constant bias $f_{0}$. In particular, ANM emerges over the range $0<D_{ac}<0.01$ and $0<f_{0}<0.3$ for the AOUP active noise considered here, illustrating that the statistical properties of the active fluctuations play an important role in determining the transport response.

We further showed that externally imposed fractional Gaussian noise substantially modifies the ANM regime. Within the range studied (H≤1/2), increasing the Hurst exponent—corresponding to reduced, not increased, anti-persistence—enlarges the ANM region and increases the maximum magnitude of the negative mobility.

Overall, these results highlight the combined influence of periodic driving, active fluctuations, and externally imposed power-law temporal correlations on directed transport in nonequilibrium systems. They provide new insight into how different sources of non-equilibrium fluctuations modify absolute negative mobility and may contribute to the design of active-particle manipulation and separation strategies in structured environments.

Finally, we emphasize that the present work is focused on the transport properties associated with absolute negative mobility in the AOUP and fractional-Gaussian-noise models retained here. A systematic investigation of complementary directions—including run-and-tumble active statistics compared at matched persistence and active diffusivity, a controlled overdamped limit of the present inertial dynamics, a properly validated treatment of quenched spatial disorder, and complementary dynamical observables such as diffusion properties, stochastic resonance, and the stability of the underlying locked and running states—represents an interesting direction for future research. 

## Figures and Tables

**Figure 1:**
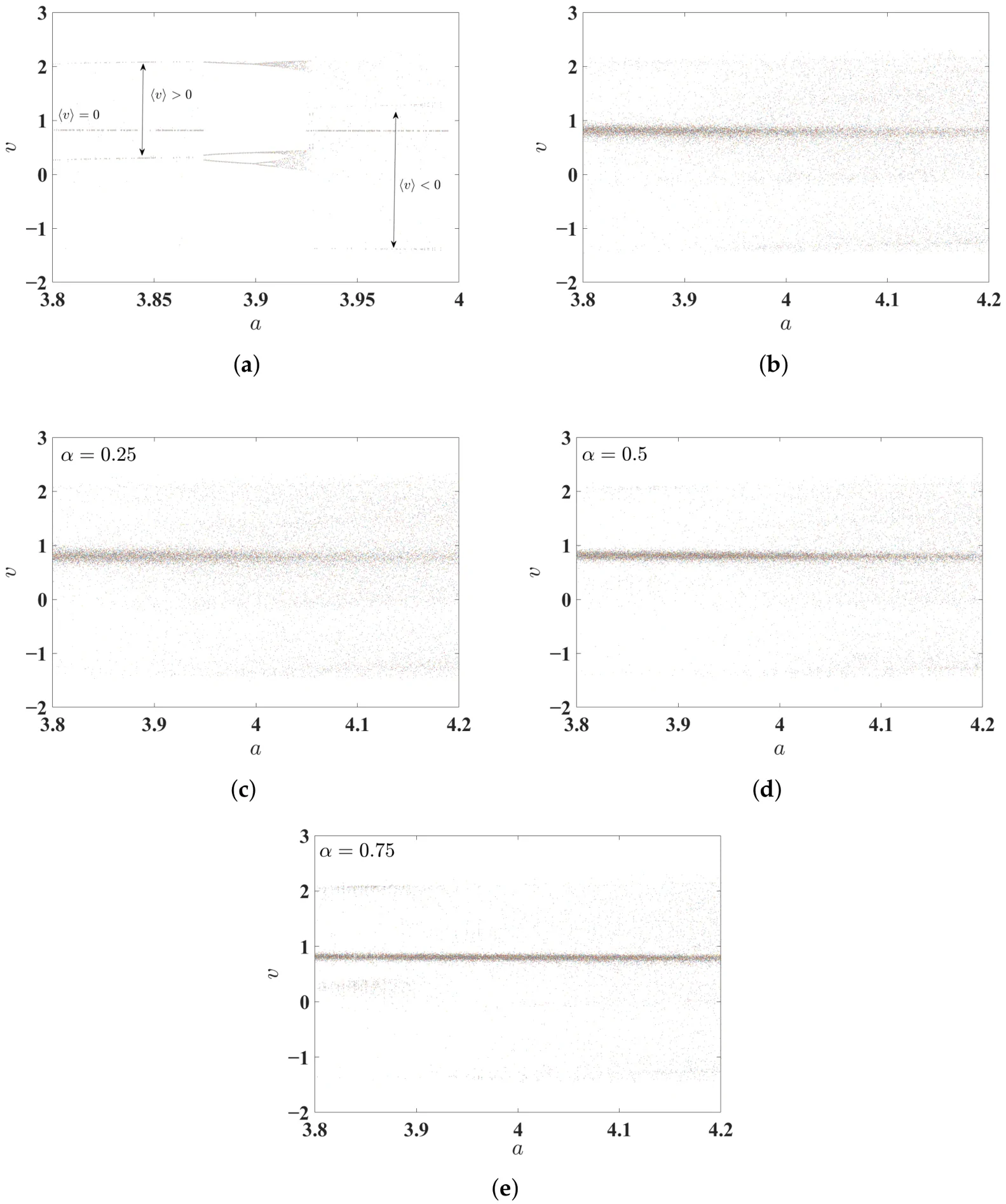
Stroboscopic velocity bifurcation diagrams for system (3) as a function of the rocking amplitude *a* of the periodic driving force acos(ωt) with ω=4.9. Panel (**a**) refers to deterministic case; (**b**) refers to white Gaussian noise (wGn); (**c**) refers to fGn with α=0.25; (**d**) refers to fGn with α=0.5; and (**e**) refers to fGn with α=0.75. The remaining parameters are γ=1.1, $D_{\alpha }=0.001$, and $f_{0}=0.15$.

**Figure 2:**
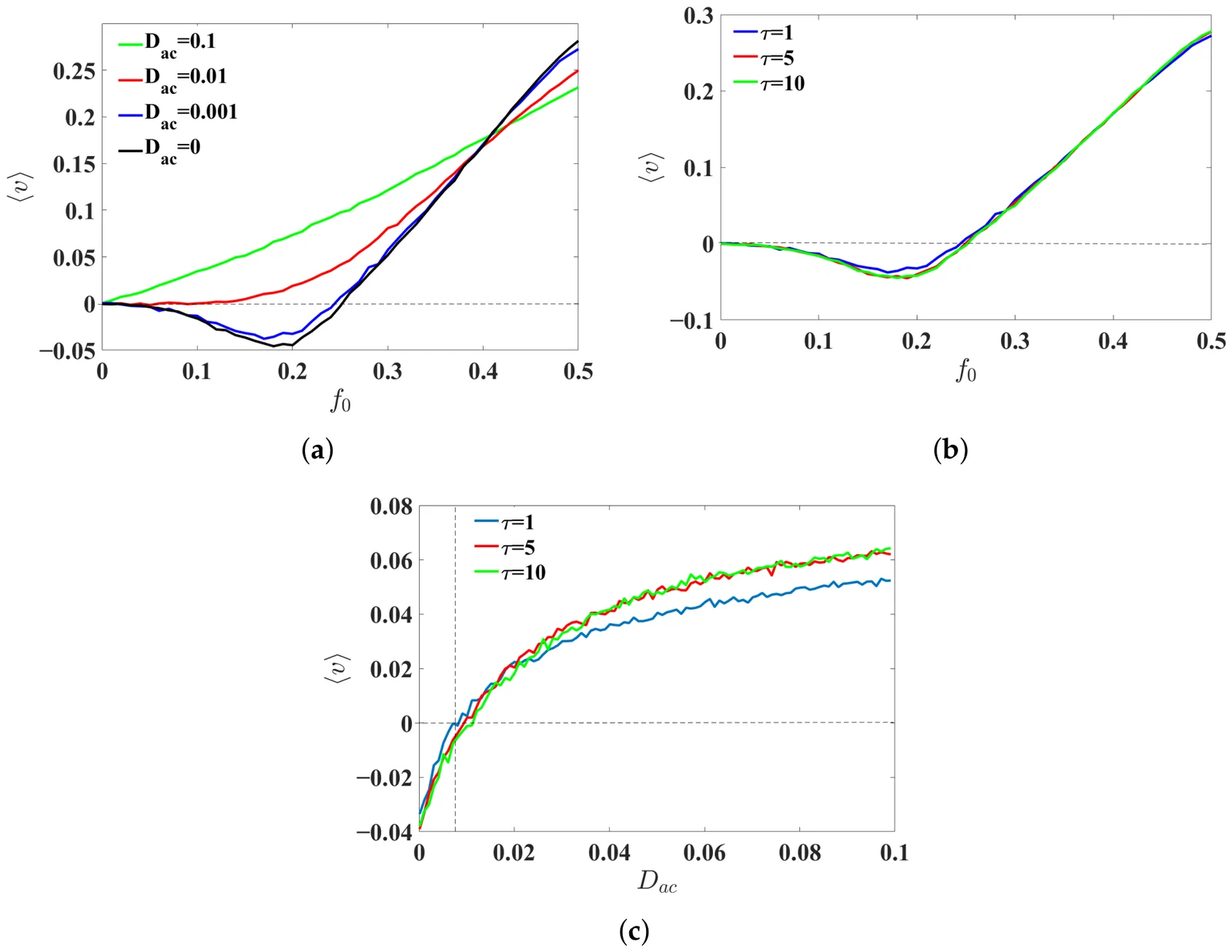
(**a**) Average velocity 〈v〉 vs. $f_{0}$ for AOUPs with wGn, τ=1 and varying $D_{ac}$, including the passive ($D_{ac}=0$) curve for reference; (**b**) $\langle v\rangle$ vs. $D_{ac}$ with varying τ and $f_{0}=0.15$, and (**c**) $\langle v\rangle$ vs. $f_{0}$ for AOUPs with $D_{ac}=0.001$ and different values of τ. The remaining parameters are a=4.2, $D_{\alpha }=0.001$, ω=4.9, and γ=1.1. Panel (**a**) illustrates that activity modulates, rather than creates, the ANM window already present at $D_{ac}=0$.

**Figure 3:**
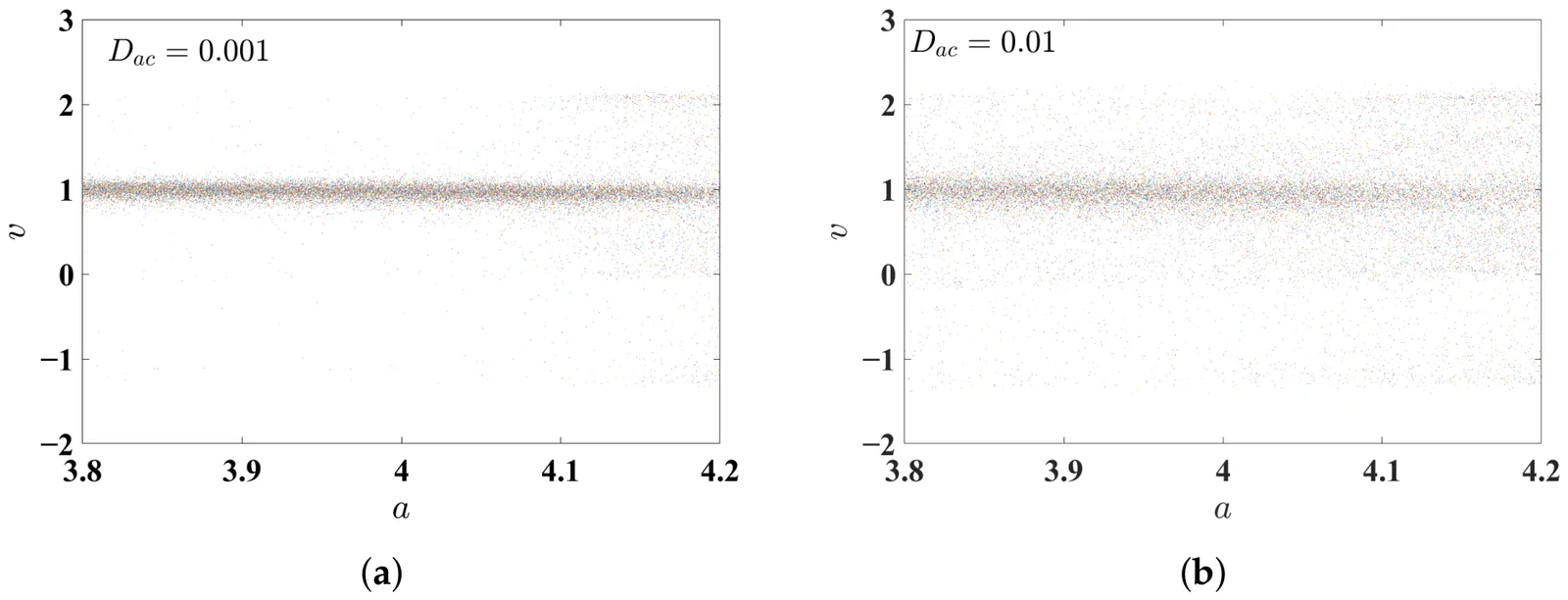

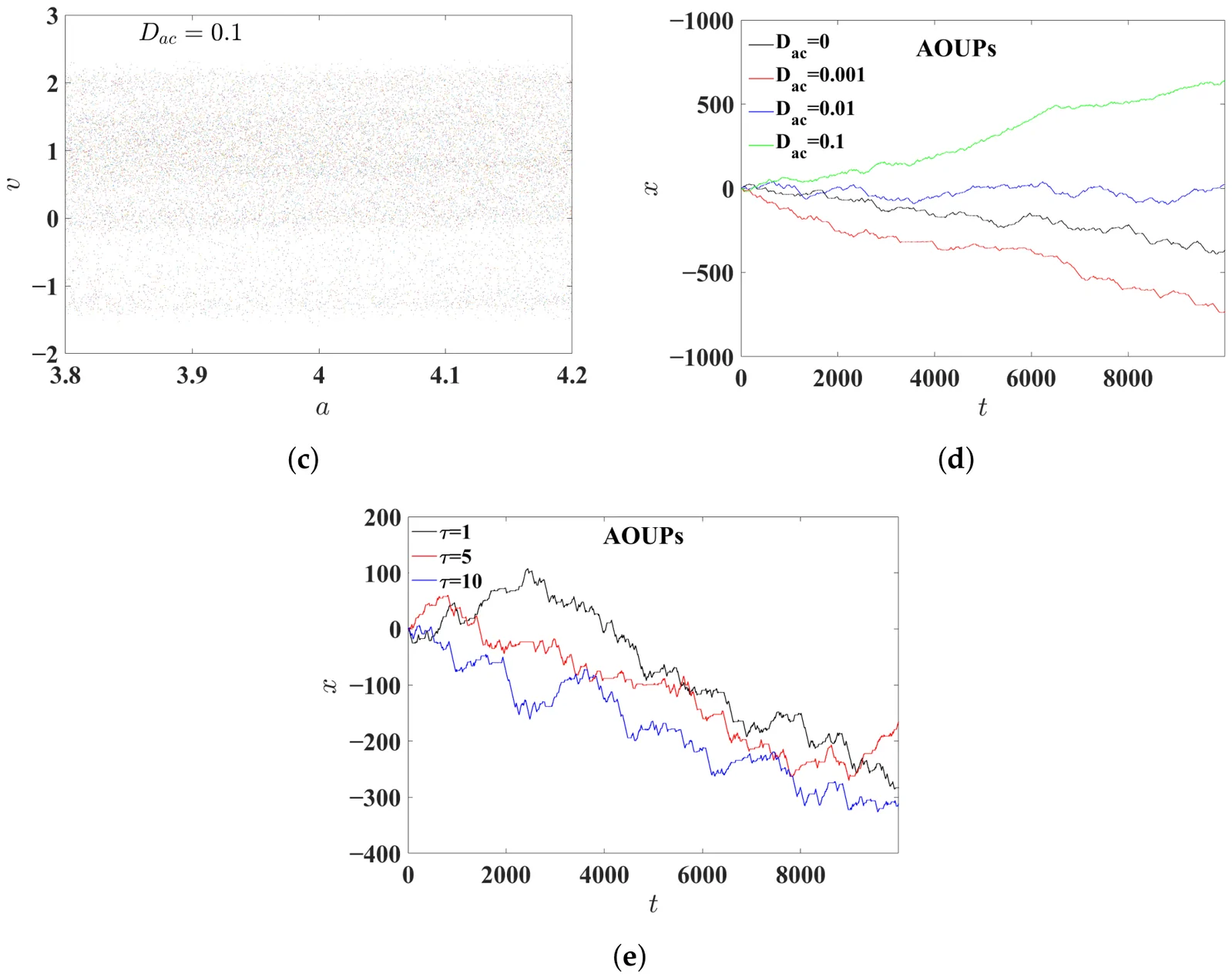
Stroboscopically sampled velocities vs. amplitude *a* and displacement *x* vs. time for AOUPs with wGn, and varying $D_{ac}$. (**a**) Velocity vs. *a* for $D_{ac}=0.001$ and τ=1; (**b**) Velocity vs. *a* for $D_{ac}=0.01$ and τ=1; (**c**) Velocity vs. *a* for $D_{ac}=0.1$ and τ=1; (**d**) Displacement trajectories for varying $D_{ac}$ at fixed τ=1; (**e**) Displacement trajectories for varying persistence time τ=1,5,10 at fixed $D_{ac}=0.001$. Fixed parameters: γ=1.1, $f_{0}=0.15$, a=4.2, ω=4.9, and $D_{\alpha }=0.001$.

**Figure 4:**
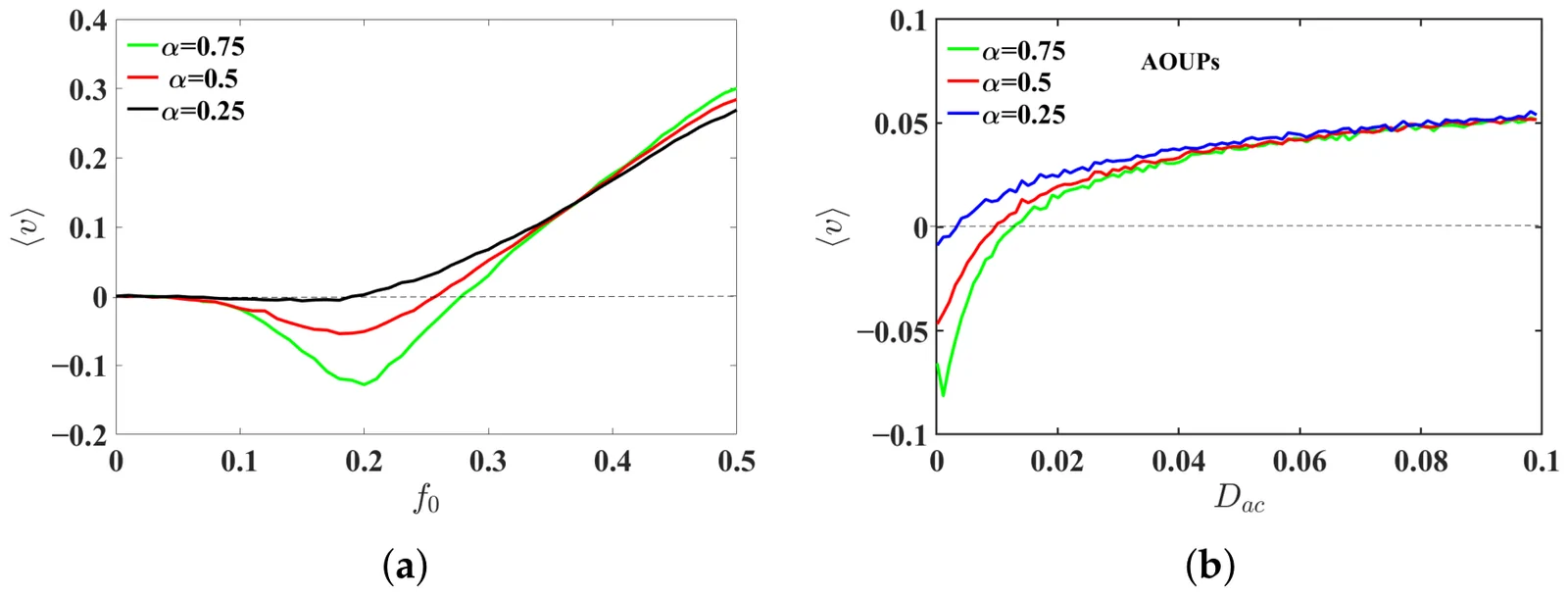
(**a**) 〈v〉 vs. $f_{0}$ for AOUPs with varying α and $D_{ac}=0.001$; (**b**) 〈v〉 vs. $D_{ac}$, varying α and $f_{0}=0.15$. The remaining parameters are a=4.2, ω=4.9, γ=1.1, $D_{\alpha }=0.001$, and τ=1.

**Figure A1:**
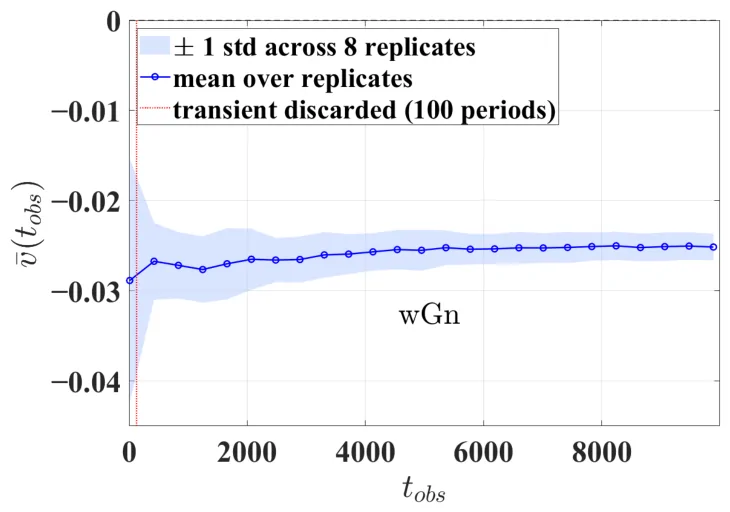
Convergence of the averaged velocity $\bar{v}\left(t_{obs}\right)$ (Equation (4)) for the wGn-plus-active-noise (AOUP) model, at $f_{0}=0.15$, a=4.2, γ=1.1, $D_{ac}=0.001$, $D_{1}=0.001$ (Δt=0.01, N=1000 trajectories per replicate, $N_{rep}=8$ independent replicates). The solid line is the mean of $\bar{v}$, computed over a growing observation window $\left[t_{tr},t_{tr}+t_{obs}\right]$, averaged across replicates; the shaded band is ±1 standard deviation across replicates. The vertical dotted line marks the transient $t_{tr}=100$ driving periods discarded before measurement. The averaged velocity reaches a stable plateau ($\bar{v}\approx -0.025$) by $t_{obs}\approx 10^{3}$, well within the total observation window of $10^{4}$, confirming that the reported value is asymptotic and not a finite-time artifact.

**Figure A2:**
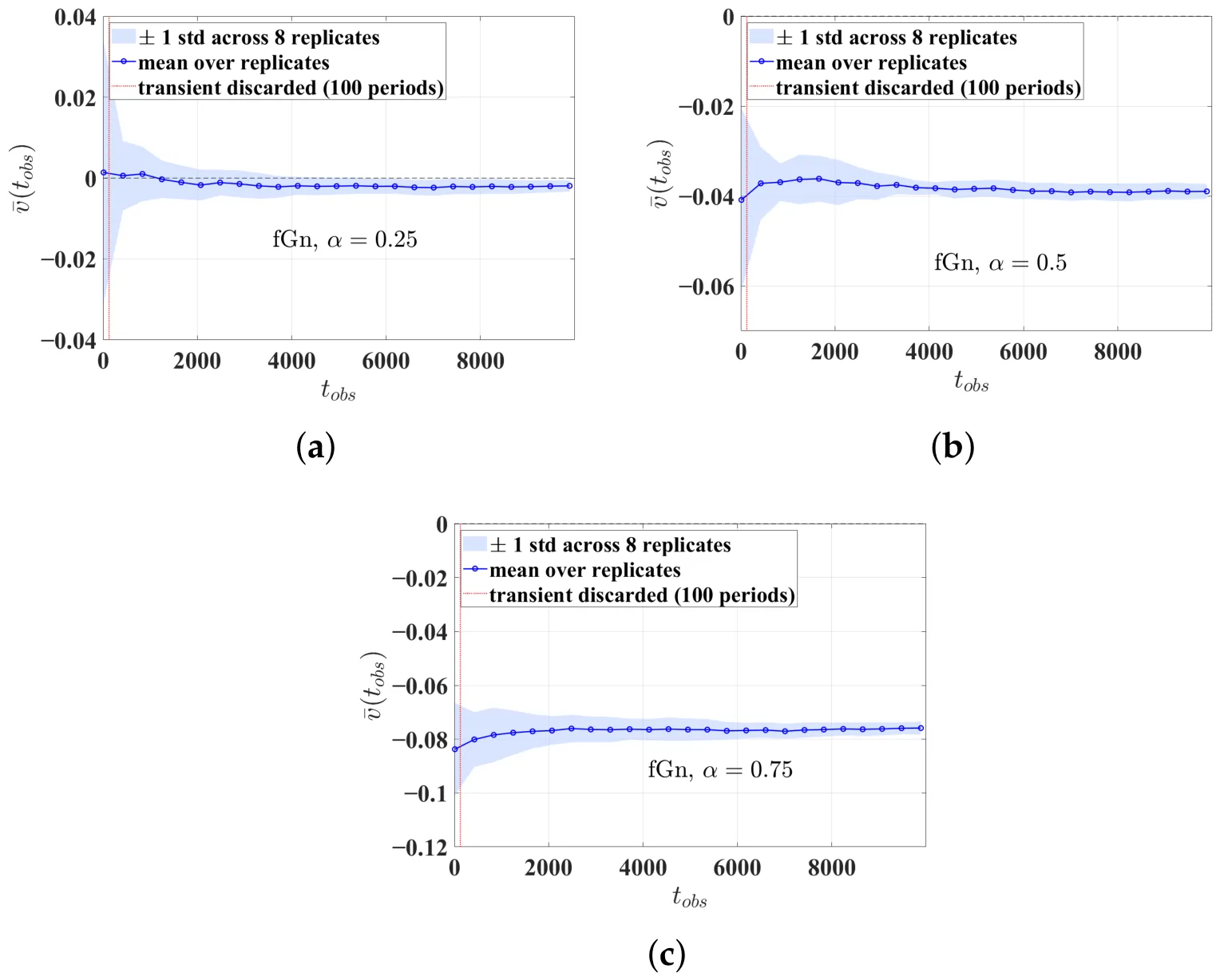
Convergence of the averaged velocity $\bar{v}\left(t_{obs}\right)$ forfractional-Gaussian-noise-plus-active-noise, at $f_{0}=0.15$, a=4.2, γ=1.1, $D_{ac}=0.001$, for (**a**) α=0.25 (H=0.125), (**b**) α=0.5 (H=0.25), and (**c**) α=0.75 (H=0.375) (Δt=0.01, N=1000 trajectories per replicate, $N_{rep}=5$–8 independent replicates). Conventions as in Figure A1. All three cases reach a stable plateau by $t_{obs}\approx 1$–$2\times 10^{3}$, with the across-replicate uncertainty band bounded away from zero at the plateau in every case, confirming that the sign of the effect is statistically well established even at the weakest value tested, α=0.25 ($\bar{v}\approx -0.0035$), and grows in magnitude with α (to $\bar{v}\approx -0.077$ at α=0.75).

**Figure A3:**
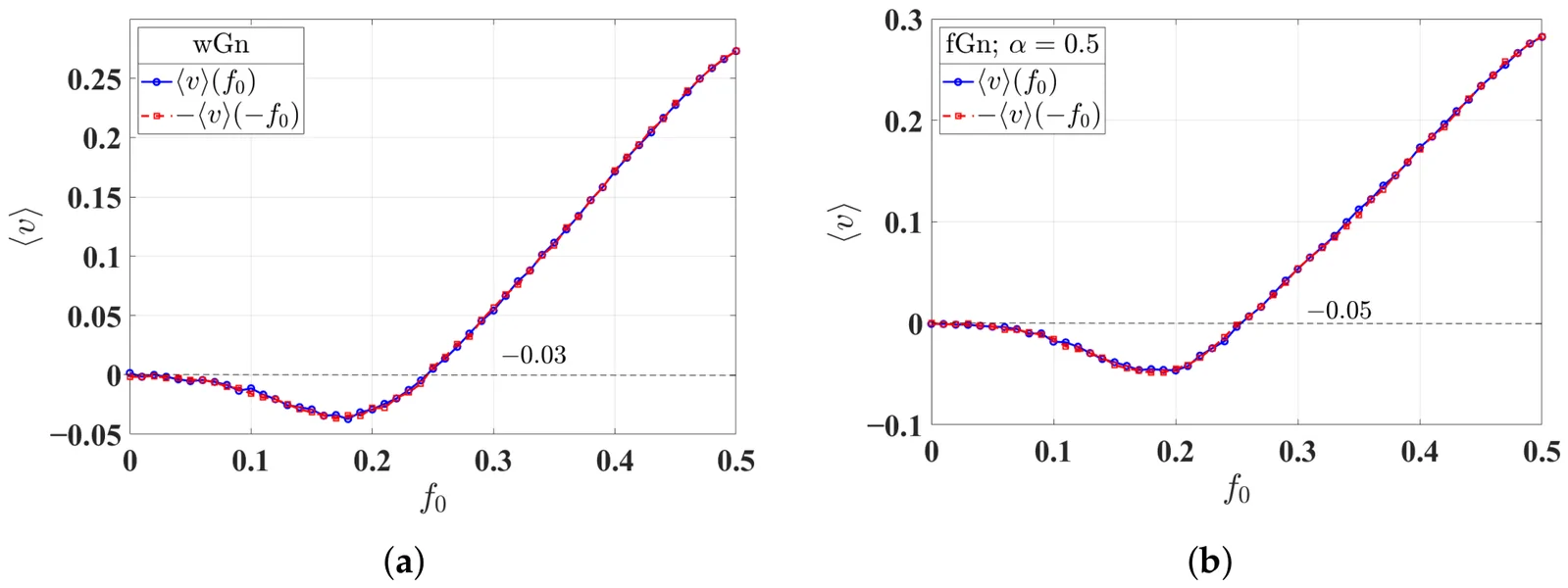
Symmetry check for the system (3) with $D_{ac}=0.001$, τ=1, a=4.2, γ=1.1, ω=4.9, $D_{H}=0.001$. Panel (**a**) corresponds to the case of wGn, while panel (**b**) corresponds to the case of fGn with α=0.5. The period-averaged velocity $\langle v\rangle \left(f_{0}\right)$ (blue) is overlaid with $-\langle v\rangle \left(-f_{0}\right)$ (red) across $f_{0}\in \left[-0.5,0.5\right]$, computed after discarding the same $t_{tr}=100$-period transient used throughout this work (Table A1). The two curves coincide to within statistical uncertainty across the full range, including the ANM region ($f_{0}\approx 0.15$–0.2), confirming the expected antisymmetry $\langle v\rangle \left(-f_{0}\right)=-\langle v\rangle \left(f_{0}\right)$ required by the spatial symmetry of the system (3).

## Data Availability

The data presented in this study are available on request from the corresponding author. The data are not publicly available because they were generated through numerical simulations and are not currently deposited in a public data repository.

## Appendix A. Numerical Methods

All simulations were performed in MATLAB 2021b. Writing Equation (3) as the first-order system $\dot{x}=v$, $\dot{v}=-\gamma v-{\tilde{V}}^{\prime }\left(x\right)+a\cos\left(\omega t\right)+f_{0}+\xi_{ac}\left(t\right)+\sqrt{2\gamma D_{\alpha }}\;\xi_{\alpha }\left(t\right)$, the velocity is advanced by a single explicit Euler–Maruyama step, in which *every* term on the right-hand side—including the friction term −γv—is evaluated at the old state $\left(x_{l-1},v_{l-1},t_{l-1}\right)$; no implicit or corrector step is used for the velocity. The position is then reconstructed from the two neighboring velocity values using the trapezoidal rule:

(A1) $$\begin{array}{cc} v_{l} & =\left(1-\gamma \;\Delta t\right)\;v_{l-1}+\Delta t\left[-2\pi \cos\left(2\pi x_{l-1}\right)+\xi_{ac}^{l-1}+a\cos\left(\omega \left(l\;-\;1\right)\Delta t\right)+f_{0}\right]+\xi_{\alpha }^{l-1}, \end{array}$$

(A2) $$\begin{array}{cc} x_{l} & =x_{l-1}+\frac{\Delta t}{2}\left(v_{l-1}+v_{l}\right), \end{array}$$

where $\xi_{ac}^{l}$ is the discretized active-noise process (Section 2) and $\xi_{\alpha }^{l}$ is the discretized noise increment associated with $\xi_{\alpha }\left(t\right)$ in Equation (3). We emphasize that Equation (A1) is an *explicit* Euler–Maruyama update: writing $\left(1-\gamma \Delta t\right)v_{l-1}=v_{l-1}-\gamma v_{l-1}\Delta t$ makes clear that the friction term uses the old velocity $v_{l-1}$, not the updated $v_{l}$, and is therefore not a backward (implicit) discretization of friction. Only the position update, Equation (A2), uses an average of the old and new velocities; this trapezoidal step improves the accuracy of the displacement (and hence of 〈v〉, Equation (5)) but does not alter the explicit nature of the velocity integration itself, since $v_{l}$ has already been computed from Equation (A1) using only quantities known at $t_{l-1}$.

The active-noise term $\xi_{ac}^{l}$ is obtained from the explicit Euler–Maruyama discretization of the Ornstein–Uhlenbeck process, Equation (2):

(A3) $$\xi_{ac}^{l}=\left(1-\frac{\Delta t}{\tau }\right)\xi_{ac}^{l-1}+\frac{\sqrt{2D_{ac}\;\Delta t}}{\tau }\;\zeta^{l-1},\;\zeta^{l-1}∼N\left(0,1\right)\;i.i.d.$$

For the noise term $\xi_{\alpha }^{l}$ we derive the discrete increment directly from Equation (3) rather than positing it. Since $\sqrt{2\gamma D_{\alpha }}\;\xi_{\alpha }\left(t\right)$ enters the velocity equation additively, its contribution over one time step is the integral $\sqrt{2\gamma D_{\alpha }}\int_{t_{l-1}}^{t_{l}}\xi_{\alpha }\left(s\right)\;ds=\sqrt{2\gamma D_{\alpha }}\;\Delta B_{\alpha ,l-1}$, where $\Delta B_{\alpha ,l-1}=B_{\alpha }\left(t_{l}\right)-B_{\alpha }\left(t_{l-1}\right)$ is an increment of fractional Brownian motion (fBm) with Hurst exponent H=α/2, since $\xi_{\alpha }\left(t\right)\equiv {\dot{B}}_{\alpha }\left(t\right)$ is fGn by definition. Because fBm increments over a step of size Δt have standard deviation scaling as ${\left(\Delta t\right)}^{H}$ rather than ${\left(\Delta t\right)}^{1/2}$, we write $\Delta B_{\alpha ,l-1}={\left(\Delta t\right)}^{H}b_{\alpha }^{l-1}$, where $\left\{b_{\alpha }^{l}\right\}$ is a unit-variance discrete-time fGn sequence with autocorrelation matching $\langle \xi_{\alpha }\left(t_{1}\right)\xi_{\alpha }\left(t_{2}\right)\rangle$ (Section 2), generated using the FFGN algorithm [82,83,84]. The term appearing in Equation (A1) is therefore

(A4) $$\xi_{\alpha }^{l-1}\;=\;\sqrt{2\gamma D_{\alpha }}\;{\left(\Delta t\right)}^{H}\;b_{\alpha }^{l-1},$$

which carries the same $\sqrt{\gamma }$ prefactor that multiplies $\xi_{\alpha }\left(t\right)$ in Equation (3); unlike the white-noise and active-noise terms, this prefactor multiplies the increment directly rather than being absorbed into an overall $\sqrt{\Delta t}$ scaling, precisely because H≠1/2 for α<1. For white noise (α=1, H=1/2), Equation (A4) reduces to the familiar Euler–Maruyama form $\xi_{1}^{l-1}=\sqrt{2\gamma D}\;\Delta W_{l-1}$ with $\Delta W_{l-1}∼N\left(0,\Delta t\right)$, consistent with Equation (A4) at H=1/2.

The integration time step is Δt=0.01 for the thermal-noise, active-noise (AOUP), and pure fractional-Gaussian-noise simulations (confirmed at production scale, $t_{max}=10^{4}$). Ensemble averages are computed over N=1000 independent trajectories per parameter value. For each noise model, the transient time $t_{tr}$ discarded before measurement, and the observation window MT (an integer number of driving periods) over which Equation (5) is evaluated, were determined from the convergence analysis shown in Figure A1 and Figure A2. Convergence is deemed reached when the averaged velocity changes by less than 5% between the second and final quarters of the observation window, and the across-replicate uncertainty band (built from $N_{rep}$ independent reruns with different noise realizations) has stabilized. Table A1 summarizes the resulting parameters for each noise model.

**Table A1:** Simulation and convergence parameters by noise model.

Noise Model	Δt	$t_{\max}$ (Steps)	$t_{\mathrm{tr}}$ (Periods)	Converged $\bar{v}$ (Example Point)
Thermal + AOUP ($f_{0}=0.15$, $D_{ac}=0.001$)	0.01	$10^{4}$ ($10^{6}$)	100	−0.025±0.002
Pure fGn, α=0.25 (H=0.125)	0.01	$10^{4}$ ($10^{6}$)	100	−0.0035±0.0006
Pure fGn, α=0.5 (H=0.25)	0.01	$10^{4}$ ($10^{6}$)	100	−0.038±0.003
Pure fGn, α=0.75 (H=0.375)	0.01	$10^{4}$ ($10^{6}$)	100	−0.077±0.005

As a further check on the definition of ANM given in Section 2 (Equation (6)), Figure A3 verifies the antisymmetry $\langle v\rangle \left(-f_{0}\right)=-\langle v\rangle \left(f_{0}\right)$ (Equation (7)) for the system (3).
